# Spatial Variations of Indoor Air Chemicals in an Apartment Unit and Personal Exposure of Residents

**DOI:** 10.3390/ijerph182111511

**Published:** 2021-11-01

**Authors:** Hironari Sakamoto, Shigehisa Uchiyama, Tomohiko Isobe, Naoki Kunugita, Hironao Ogura, Shoji F. Nakayama

**Affiliations:** 1 Faculty and Graduate School of Engineering, Chiba University, Chiba 263-8522, Japan; hironari-sakamoto@chiba-u.jp (H.S.); hiro_ogura@faculty.chiba-u.jp (H.O.); 2 Japan Environment and Children’s Study Programme Office, National Institute for Environmental Studies, Ibaraki 305-8506, Japan; isobe.tomohiko@nies.go.jp (T.I.); fabre@nies.go.jp (S.F.N.); 3 School of Health Sciences, University of Occupational and Environmental Health, Fukuoka 807-8555, Japan; kunugita@med.uoeh-u.ac.jp

**Keywords:** personal exposure, indoor air quality, spatial variation, diffusive sampler

## Abstract

Indoor air quality (IAQ) can greatly affect health in people spending much time indoors. However, the influence of IAQ on personal exposure to chemical compounds in Japan remains poorly investigated. Hence, this study aimed to clarify this influence thoroughly within one apartment. We surveyed the concentrations of 61 chemical compounds in the air in nine different spaces within an apartment unit, as well as the personal exposure of two residents in Japan. Using three kinds of diffusive samplers, this study was conducted continuously for 7 days in summer and winter. Health risks were evaluated by calculating the margin of exposure (MOE) using the measured concentrations. Some chemical concentrations showed large spatial variations and the personal exposure concentrations of these compounds also differed among residents. According to the calculated MOE, the chemicals with the highest health risk were acrolein, *p*-dichlorobenzene, and acetaldehyde in summer and acrolein, nitrogen dioxide, formic acid, *p*-dichlorobenzene, and benzene in winter. The IAQ of the house could be divided in two, and the IAQ in the space where residents spent much time (i.e., bedroom) highly affected each of the residents’ exposure. Investigating chemical concentrations in multiple spaces (including bedroom and living room) is necessary to understand the effect of IAQ on personal exposure.

## 1. Introduction

Indoor air contains numerous chemicals emitted from building materials and consumer products. The concentrations of these chemicals in indoor air tend to be higher than those in outdoor air, owing to its closed environment. Nowadays, the airtightness and heat insulation of houses have been improved to save energy; however, if the area is not properly ventilated, chemicals are more likely to accumulate in indoor air. Given that many people spend 90% of the day indoors [1], indoor air quality (IAQ) can have a large effect on individual health. IAQ may cause not only sick building syndrome but also chronic diseases caused by long-term indoor air exposure.

The World Health Organization (WHO) [2,3], as well as many countries, has developed guideline values for indoor air pollutants. In Japan, the Ministry of Health, Labor, and Welfare (MHLW) has developed IAQ guidelines for 13 chemicals (formaldehyde, toluene, xylene, *p*-dichlorobenzene, ethylbenzene, styrene, chlorpyrifos, di-*n*-butyl phthalate, tetradecane, di-(2-ethylhexy)phthalate, diazinone, acetaldehyde, and fenobucarb) [4]. However, over time, the building materials and consumer products used have changed, thereby also changing the kinds and concentrations of chemicals in indoor air [5]. Thus, to understand health risks, IAQ should be continuously investigated by evaluating a wide range of chemicals, including those that are not yet included in the guideline.

In Japan, although IAQ has already been widely surveyed [6,7,8,9,10,11], few studies have investigated how IAQ influences personal exposure, which should be measured for accurate risk assessment. In other countries, some studies assessing volatile organic compounds (VOCs) were conducted to investigate the personal exposure concentrations of residents as well as the indoor air concentrations of houses [12,13,14]. Personal exposure concentrations were found to be strongly associated with indoor air concentrations because residents spent much time at home. In these studies, only one or two spaces in each house were used to measure indoor air concentration for investigating a large number of samples. However, even within the same house, chemical concentrations can be greatly different between spaces depending on its situation, such as where emission sources and air vents are located.

Given these insights, this study aimed at the following: (1) to reveal spatial variations of chemical concentrations in one apartment unit and the personal exposure concentrations of the residents; (2) to determine the high-risk indoor air chemicals in the house according to the personal exposure concentrations and to presume their emission sources; (3) to clarify the space where personal exposure was greatly affected by IAQ and to estimate the personal exposure concentrations according to the IAQ. Thus, we measured the concentrations of 61 chemical compounds—namely, 40 VOCs, 15 carbonyl compounds, 5 acidic gases, and ozone—in nine spaces in an apartment unit and the personal exposure for the residents concurrently.

## 2. Materials and Methods

### 2.1. House Characteristic

**Figure 1:**
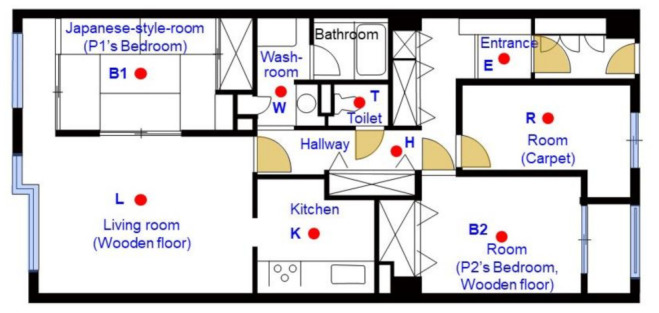
Floor plan of the investigated house. Red closed circles with characters show sampling points.

Measurements were performed inside an apartment house with 81.44 m^2^ floor area on the fourth floor of a 13-story steel-framed, reinforced concrete building, which was completed in August 1987, in Chiba-shi, Japan. This type and size of apartment are representative of apartments in Japan. According to the 2018 Housing and Land Survey of Japan [15], the average floor area and number of rooms for apartment houses are 75.11 m^2^ and 3.88, respectively, and 72% of apartments have the same structure. Figure 1 shows the house’s floor plan. The chemical concentrations in indoor air in nine spaces of the house and in the personal air of the two residents (P1, P2) were investigated. Each investigated space was labeled as the first letter of the room name, as shown in Figure 1 (e.g., living room, P1’s bedroom, and P2’s bedroom were labeled as L, B1, and B2, respectively). The floor of room B1 was covered by tatami, which is a Japanese traditional mat made of rushes.

### 2.2. Sampling and Analysis

We used three kinds of diffusive sampling devices (DSDs)—namely, DSD-CX packed with Carboxen 572 particles for measuring VOCs, DSD-BPE/DNPH [16] packed with 2,4-dinitrophenyl hydrazine (DNPH) and *trans*-1,2-bis(2-pyridyl) ethylene (BPE) coated silica for measuring ozone and carbonyl compounds, and DSD-TEA packed with triethanolamine (TEA) for measuring acidic gases. These devices have an exposure component made of a porous sintered polyethylene (diffusion filter) and an absorbent component, which is different among such devices. The surface area and thickness of the diffusion filter were 3.93 cm^2^ and 1 mm, respectively. 

An overview of the procedure from sampling to analysis is shown in Figure S1 in the Supplementary Materials. The continuous 7-day samplings for the nine spaces and two residents were conducted simultaneously in two seasons: summer (19–26 July 2020) and winter (19–26 March 2021). All the devices had the same measuring procedures, except for elution and analysis. After taking out the sampler from a heat-sealed aluminum plastic-laminated sachet, we removed the glass shelter tube and oriented the device vertically. At this point, sample exposure began. After 7 days, sampling was stopped by replacing the shelter tube. Then, we repacked the device in an aluminum-laminated bag and refrigerated it at 4 °C (organic solvent-free). For the elution and analysis of each device, we followed the procedure of our previous study [10]. The apparatuses used for analysis in this study were a gas chromatography-mass spectrometry (GC/MS) system (QP 2010 Ultra, Shimadzu, Kyoto, Japan) for VOCs, a high-performance liquid chromatography (HPLC) system (Prominence LC-20, Shimadzu, Kyoto, Japan) for ozone and carbonyls, and an ion chromatography (IC) system (DionexICS-2100 Integrated Reagent-Free, Thermo Fisher Scientific, Waltham, MA, USA) for acidic gases. Indoor temperature and humidity were monitored in one space using a data logger (TR-72Ui, T&D Co., Nagano, Japan) at 30 min intervals. Furthermore, we requested the residents to record their time spent staying at home.

### 2.3. Calculation

The concentrations were calculated using the following equation:

*C* = *WR*^−1^*t*^−1^ × 10^6^ (1)

where *C* is the concentration of the target chemical (μg/m^3^), *W* is the amount of absorbate (μg), *R* is the sampling rate (mL/min), and *t* is the sampling time (min). *W* was determined from each integrated area of the chromatogram. The *R* value for each chemical was determined according to the procedure previously reported by Uchiyama et al. [17]. Briefly, parallel measurements were performed using an active sampling method for 7 days, and the amounts collected by diffusive sampling were compared with those collected by active sampling. Then, *R* was obtained from the slope of the regression line of this comparison graph.

### 2.4. Risk Characterization

The health risks for measured concentrations were characterized using the margin of exposure (MOE) approach described comprehensively by Azuma et al. [18]. MOE was calculated from the following equation. A lower MOE indicates a higher risk.

MOE = RfC/*C* (2)

where RfC is the inhalation reference concentration, and *C* is the indoor air concentration or personal exposure concentration measured in this study. The RfC for each chemical was determined by Azuma et al. on the basis of WHO air quality guidelines or the estimated no observed adverse effect level (NOAEL) (or the lowest observed adverse effect level (LOAEL)) from documents or reports published by international and national agencies for noncancer effects [18]. For cancer effects, RfCs were described as concentrations associated with an excess lifetime carcinogenic risk of 1/100,000 [18]. In this study, the same RfCs determined by Azuma et al. were used.

## 3. Results

Table 1 summarizes the indoor air, outdoor air, and personal exposure concentrations for selected chemicals. Overall, almost all indoor concentrations were higher than outdoor concentrations except for ozone. Personal exposure was lower than the maximum potential exposure from indoor air concentrations in different spaces. For some chemicals, the personal exposure concentrations were largely different between P1 and P2. The personal exposure concentrations of acetaldehyde (summer), tetradecane (summer), styrene (winter), formic acid (winter), and nitrogen dioxide (winter) were higher in P1 than in P2 by a factor of 3.6, 3.1, 5.5, 3.3, and 4.6, respectively. Meanwhile, the exposure concentration of *p-*dichlorobenzene (summer and winter) was higher in P2 than in P1, by a factor of 4.3 and 4.6, respectively.

**Figure 2:**
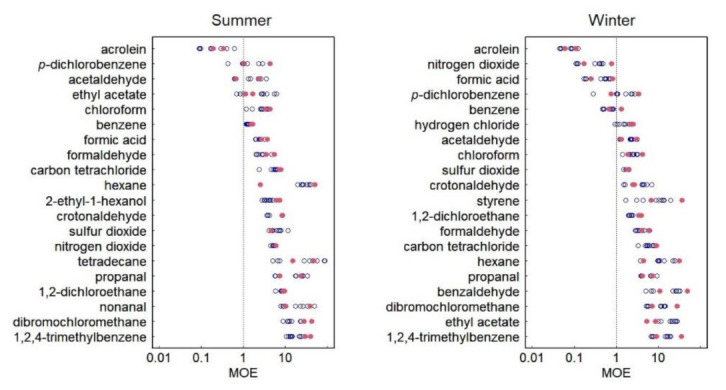
Distributions of MOE for 20 chemicals with the lowest MOE values in summer and winter. Blue open circles indicate data on indoor air, and red closed circles indicate data on personal exposure.

Figure 2 shows the distributions of MOE calculated from the measured concentrations. In both seasons, the chemicals were vertically arranged according to the minimum MOE. The lower the MOE, the higher the health risk. An MOE of <1 indicated that the measured concentration was higher than the RfC. In this study, the chemicals with an MOE of ≤1 for personal exposure had the highest health risk. These chemicals were acrolein, *p-*dichlorobenzene, and acetaldehyde in summer, and acrolein, nitrogen dioxide, formic acid, *p-*dichlorobenzene, and benzene in winter.

**Figure 3:**
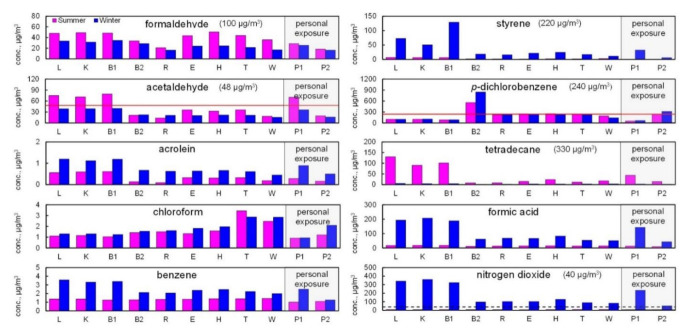
Concentrations of chemical compounds in various indoor air spaces and personal exposure. Guideline values by MHLW and WHO (presented in parentheses) are shown as solid and dashed lines, respectively.

**Figure 4:**
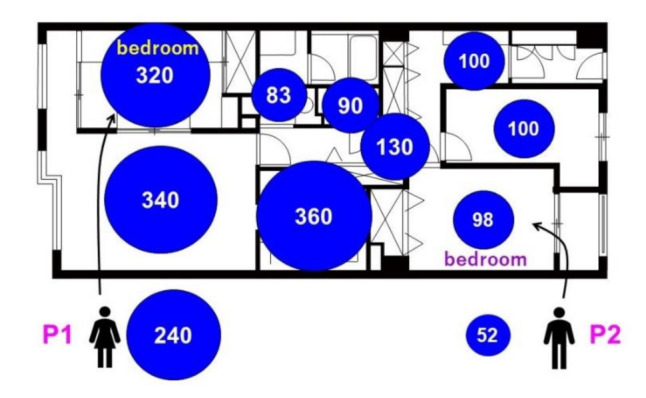
Concentrations of nitrogen dioxide in winter with the floor plan. Each circle area and number shows the concentration (μg/m^3^) in the space or the resident.

Figure 3 shows the concentrations of chemicals that showed high risks or were emitted substantially by the source for each space and resident. As an example, concentrations of nitrogen dioxide in winter are also shown more schematically with the floor plan in Figure 4. Other compounds are shown in Figure S2 in the Supplementary Materials. The mean concentrations of formaldehyde and tetradecane were higher in summer than in winter by a factor of 1.6 and 13, respectively. Conversely, the mean concentrations of acrolein, benzene, styrene, formic acid, and nitrogen dioxide were higher in winter than in summer, by a factor of 2.3, 1.9, 11, 7.0, and 24, respectively. Additionally, Figure 3 shows distinctive spatial variations for some chemicals. The mean concentrations of chemicals in L, K, and B1 were higher than those in other spaces, especially for acetaldehyde and tetradecane in summer, and acrolein, benzene, styrene, formic acid, and nitrogen dioxide in winter (by a factor of 2.9, 7.7, 1.9, 1.6, 4.6, 3.1, and 3.4, respectively). The mean concentrations of chloroform in both summer and winter were higher in T and W than in other spaces by a factor of 2.3 and 1.8, respectively. Additionally, the mean concentration of *p-*dichlorobenzene in both summer and winter was higher in B2 than in other spaces by a factor of 3.1 and 5.0, respectively.

The similarity of the compositions at each space and personal exposure was evaluated using Pearson’s correlation coefficient between each chemical concentration dataset (Table 2). The data of L, K, B1, and P1, as well as those of the other spaces and P2, were highly correlated.

Each personal exposure concentration of the highest-risk chemicals was roughly estimated by multiplying the concentration in the bedroom and the percentage of time spent at home. P1 and P2 spent 66% and 55% of their time at home in summer, and 87% and 54% in winter, respectively. Table 3 shows the measured and estimated personal exposure concentrations of the highest-risk chemicals. The ratios of estimated to measured concentrations were 0.44–1.4. The estimation of P1’s exposure in winter showed good agreement. Meanwhile, P2’s exposure concentrations of *p-*dichlorobenzene were overestimated, whereas those of acrolein and acetaldehyde were underestimated.

## 4. Discussion

### 4.1. Spatial Variations and Personal Exposure

This study revealed spatial variations of chemical concentrations in an apartment unit and the personal exposure concentrations of the residents as well as the outdoor air concentrations in Japan (Table 1). The personal exposure concentrations of several chemicals were greatly different between P1 and P2. For example, the personal exposure concentration of styrene (winter) was higher in P1 than in P2 by a factor of 5.5, while that of *p-*dichlorobenzene (winter) was higher in P2 than in P1 by a factor of 4.6. For such chemicals, indoor air concentrations also differed largely between spaces in the same house. It suggests that indoor air concentration in a certain space cannot represent that in the house. Moreover, the personal exposure concentrations were generally lower than the maximum indoor air concentrations in the house and higher than the outdoor air concentrations except for ozone. The personal exposure concentration depends on concentrations in all the spaces where the person stays, including outside the house, and on the time spent in each of the spaces. Thus, these results indicate that the IAQ of the apartment unit was one of the environments that greatly affected personal exposure and that the influence of the outdoor air quality was rather small. However, as an exception, the exposure concentrations of hexane in summer and toluene in winter for P2 were substantially higher than the indoor air concentrations, probably because the workplace of P2 was a laboratory, where such solvents were often used. In addition, except for such chemicals, there was a trend that the higher the concentration in air of the bedroom, the higher the personal exposure concentration. It indicated that the personal exposure concentrations were seemingly influenced by the IAQ of the bedroom, where the resident spent a long time. Considering the above discussion, the personal exposure concentrations are likely to reflect the lifestyle of each person. Therefore, it is necessary to measure personal exposure concentration to evaluate it accurately.

Table 4 compares the concentrations of selected chemicals between this study and previous studies [10,19,20,21]. While our study results present the minimum and maximum concentrations in an apartment, the results of other studies are medians or geometric means calculated from each survey conducted in many houses. As for the other studies in Table 4, almost all of the indoor samplings were carried out only in living rooms. Except for styrene and *p-*dichlorobenzene, the minimum concentrations in this study were comparable with those in New York and Japan. Meanwhile, the maximum concentrations of formaldehyde, acetaldehyde, and chloroform were higher in our study than in the other studies, particularly for acetaldehyde in summer by a factor of >4. Conversely, benzene concentrations were lower than those in Beijing. Moreover, the concentrations of styrene (in winter) and *p-*dichlorobenzene were considerably higher in our study than in the other studies because this apartment had strong emission sources for these compounds. In particular, the maximum concentrations of styrene (in winter) and *p-*dichlorobenzene were higher by 2 and 1 order of magnitude, respectively. Apart from styrene and *p-*dichlorobenzene, the spatial variations of formaldehyde and acetaldehyde in the apartment were comparable with or greater than the variations of median values between cities.

### 4.2. Risk Characterization and Emission Sources

According to the MOE for the personal exposure, the chemicals with the highest health risk were acrolein, *p-*dichlorobenzene, and acetaldehyde in summer and acrolein, nitrogen dioxide, formic acid, *p-*dichlorobenzene, and benzene in winter (Figure 2). Azuma et al. assessed the health risk of 49 indoor air pollutants in 602 houses throughout Japan [18]. They reported that the highest-risk pollutants were acrolein, nitrogen dioxide, benzene, formic acid, and hydrogen chloride. In addition, propanal, acetaldehyde, and *p-*dichlorobenzene were recognized as high-risk pollutants. The highest-risk chemicals found in this study were nearly in line with those in this previous study.

Spatial variations of high-risk or substantially emitted chemicals are illustrated in detail in Figure 3 and Figure 4. According to this figure, we concluded that each emission source in the house was as follows.

1. Formaldehyde was detected at a certain level in every space, possibly because its emission source was building materials, which were ubiquitous inside the apartment.
2. Acetaldehyde and some VOCs, such as limonen, hexanal, and nonanal, are emitted by wooden materials [22]. Their concentrations were all higher in L, K, and B1 than in the other spaces. Additionally, acetaldehyde can be produced easily by the hydrolysis of vinyl acetate monomer in vinyl acetate polymers [23]. The concentration of acetic acid, which can also be produced by such hydrolysis, was also higher in L, K, and B1. Therefore, acetaldehyde was emitted by both sources.
3. Acrolein is produced by heating activities and emitted by wood products [24]. Its emission from the latter source is expected to increase in summer; thus, a higher concentration in winter was possibly attributed to the use of a kerosene fan heater at L.
4. Chloroform in indoor air is emitted from chlorinated water [25], and its concentration can be higher in the space where a large amount of water is used (e.g., shower, bath, and toilets). In addition, the estimated indoor source emission rates for chloroform were similar between seasons in New York City and Los Angeles [19]. Although the source emission rates were not investigated in this study, the concentrations showed little seasonal variation, consistent with the previous study.
5. Benzene is emitted by indoor heating activities, such as gas heating [26], cooking [27], incense burning [28], and smoking [29]. Thus, the higher concentration in winter possibly resulted from using a kerosene fan heater in L.
6. In winter, styrene concentrations were higher in L, K, and B1 than in other spaces. Its emission source could be the polystyrene bead cushion, which was purchased in February 2021, placed in B1.
7. The major emission source of indoor *p-*dichlorobenzene is a moth repellent [30]. Therefore, the higher concentration in B2 was probably attributed to the mothballs placed in B2.
8. In summer, tetradecane concentrations were higher in L, K, and B1 than in other spaces, and the emission source seemed to be the electronic mosquito repellent placed in L.
9. In winter, formic acid and nitrogen dioxide concentrations were higher in L, K, and B1, suggesting that the emission source was a kerosene fan heater used in L. Likewise, our previous research presented that the concentrations of these compounds were higher in houses using kerosene or gas heaters [10]. Thus, the results in the present study agree with those in our previous study.

Given the results of risk characterization and the discussion of emission sources, replacing mothballs and the kerosene fan heater with other products or minimizing their amount of use should be considered to decrease the concentrations of *p-*dichlorobenzene, nitrogen dioxide, and formic acid, as well as acrolein and benzene.

### 4.3. Effect of IAQ on Personal Exposure

Pearson’s correlation revealed that the concentrations of L, K, B1 and P1, as well as those of the other spaces and P2, were highly correlated (Table 2). Thus, the IAQ of the house can be divided in two, and the personal exposure concentrations depended on where the residents spent most of the time. In particular, the IAQ of the bedroom probably had a large effect on the personal exposure concentration. The difference in IAQ of the house seemed to result from physical separation between L, K, and B1 and the other spaces by a door because of using an air conditioner or heater in L.

Subsequently, we roughly estimated each personal exposure concentration of the highest-risk chemicals according to the concentration in the bedroom and the percentage of time spent at home (Table 3). As a result, the estimated concentrations generally agreed with the measured concentrations. In particular, personal exposure concentrations for P1 in winter are almost equal to the estimated values due to staying long-time at home during the period. Meanwhile, P2’s exposure concentration of *p-*dichlorobenzene was overestimated, probably because P2 spent some time in spaces other than B2 (e.g., living room), and the concentration in that space was considerably lower than that in B2. In contrast, P2’s exposure concentrations of acrolein and acetaldehyde were underestimated, probably for the opposite reason. Therefore, to improve the accuracy of P2’s exposure concentrations, we should consider the concentrations in L (the space where P2 spends most time, except B2) and the ratio of sleeping hours.

## 5. Conclusions

First, indoor air concentrations or personal exposure concentrations of some chemicals were largely different between spaces or residents in the same house. The spatial variations in the apartment for several chemicals were at the same level as or greater than the differences in median values between cities. Second, the calculated MOE from the measured concentrations identified that the chemicals with the highest health risk were acrolein, *p-*dichlorobenzene, and acetaldehyde in summer and acrolein, nitrogen dioxide, formic acid, *p-*dichlorobenzene, and benzene in winter. The emission sources were also presumed. Third, correlation analysis revealed that the IAQ of the house was divided in two and that the personal exposure of the residents was highly influenced by the IAQ in the space where they mainly stayed (i.e., bedroom). Then, the exposure concentrations were estimated by multiplying the concentrations in the bedroom and the percentage of time spent in the apartment. Overall, the estimated concentrations nearly agreed with the measured ones. However, inaccurate estimations occurred in some cases, probably caused by spatial variations of the chemicals in the house.

Therefore, to understand the effect of IAQ on personal exposure, we need to consider spatial variations of indoor air chemicals in houses and choose multiple spaces where the resident spends a long time (including at least the bedroom and living room) for measurement. Our study revealed that the indoor air concentration varies greatly depending on the room type and that each room concentration and the stay time affect individual exposure; therefore, it is difficult to perform health risk assessments based on the results of measuring only one location in a house. Measurements in multiple locations in a house should be done, and individual exposure measurement is effective/accurate for risk evaluation.

## Figures and Tables

**Table 1:** Summary of indoor air, outdoor air, and personal exposure concentrations for selected chemicals (μg/m^3^).

	Summer							Winter						
Compound	Indoor Air				Personal Exposure		Outdoor Air	Indoor Air				Personal Exposure		Outdoor Air
	Mean (Min.–Max.)	L	B1	B2	P1	P2	O	Mean (Min.–Max.)	L	B1	B2	P1	P2	O
formaldehyde	42 (21–51)	48	48	34	29	18	1.9	26 (16–35)	34	35	29	26	16	1.5
acetaldehyde	43 (14–79)	75	79	21	71	20	1.0	27 (15–40)	39	40	23	36	16	1.9
acrolein	0.35 (0.09–0.61)	0.56	0.61	0.13	0.29	0.16	0.0	0.80 (0.45–1.2)	1.2	1.2	0.67	0.89	0.50	0.0
crotonaldehyde	0.19 (0.00–0.41)	0.35	0.41	0.00	0.18	0.0	0.0	0.49 (0.21–1.0)	1.0	0.60	0.21	0.61	0.56	0.0
chloroform	1.7 (1.1–3.4)	1.1	1.1	1.4	0.91	1.2	0.28	1.8 (1.2–2.9)	1.3	1.2	1.6	0.95	2.1	0.15
carbon tetrachloride	0.85 (0.64–1.8)	0.71	0.66	0.72	0.54	0.59	0.57	0.75 (0.54–1.3)	0.59	0.54	0.70	0.48	0.46	0.52
1,2-dichloroethane	0.20 (0.18–0.27)	0.20	0.20	0.20	0.18	0.17	0.20	0.74 (0.66–0.83)	0.68	0.66	0.77	0.46	0.40	0.2
benzene	1.4 (1.2–1.4)	1.4	1.2	1.3	1.0	1.1	1.2	2.6 (2.0–3.6)	3.6	3.4	2.1	2.5	1.3	1.1
toluene	12 (10–13)	13	12	12	7.3	7.0	2.4	8.2 (5.2–12)	12	11	6.9	8.8	24	3.6
ethylbenzene	4.2 (3.7–5.5)	4.2	3.7	4.0	2.5	2.2	0.80	7.4 (3.3–16)	12	16	4.4	9.0	2.3	1.4
*m, p-*xylene	4.1 (3.0–5.2)	3.3	3.0	4.6	2.0	2.9	0.64	7.6 (3.8–14)	14	12	4.7	10	2.9	1.2
*o-*xylene	2.3 (1.5–2.9)	1.7	1.5	2.7	0.98	1.3	0.20	3.8 (2.0–7.1)	7.1	6.3	2.3	5.1	1.3	0.30
styrene	3.5 (1.4–7.0)	7.0	6.2	1.9	1.5	0.25	0.0	40 (11–130)	73	130	18	33	6.0	0.0
*p*-dichlorobenzene	220 (84–560)	100	84	560	56	240	0.10	250 (88–850)	100	88	850	70	320	0.10
hexane	6.0 (4.1–8.3)	5.1	4.1	6.2	3.2	63	1.5	16 (6.6–44)	7.4	6.6	44	5.2	37	1.4
tetradecane	45 (7.7–130)	130	100	7.7	44	14	1.9	3.5 (1.7–5.5)	5.5	4.8	1.7	2.9	1.9	0.12
ethyl acetate	44 (12–110)	87	76	14	65	45	4.3	4.6 (2.7–7.5)	7.5	6.5	3.0	8.7	14	4.0
2-ethyl-1-hexanol	35 (26–46)	41	37	46	21	17	0.0	8.8 (6.0–12)	12	11	9.2	8.0	4.6	0.0
ozone	2.9 (0.92–5.4)	5.2	5.4	1.4	4.9	1.3	48	2.3 (0.92–4.1)	2.6	1.8	0.92	2.9	4.6	52
formic acid	15 (11–19)	18	19	11	13	9.6	3.2	110 (51–210)	190	190	62	140	43	3.2
nitrogen dioxide	7.7 (6.6–9.0)	8.0	7.5	7.7	6.8	6.6	15	180 (83–360)	340	320	98	240	52	16
sulfur dioxide	3.2 (1.7–5.0)	3.3	1.7	2.9	4.3	4.3	4.7	12 (10–14)	14	13	12	11	11	2.6

**Table 2:** Correlation coefficients between chemical concentrations dataset of each space and resident.

	L	K	B1	B2	R	E	H	T	W	P1	P2
**L**		**0.998**	**0.988**	0.338	0.650	0.640	0.719	0.605	0.730	**0.996**	0.374
**K**	**0.974**		**0.977**	0.339	0.652	0.640	0.720	0.606	0.732	**0.998**	0.376
**B1**	**0.992**	**0.979**		0.305	0.616	0.608	0.688	0.573	0.694	**0.975**	0.341
**B2**	0.503	0.535	0.484		**0.931**	**0.937**	0.895	**0.950**	0.876	0.335	**0.948**
**R**	0.549	0.584	0.535	**0.991**		**0.998**	**0.995**	**0.997**	**0.988**	0.651	**0.921**
**E**	0.603	0.638	0.597	**0.981**	**0.991**		**0.994**	**0.999**	**0.987**	0.638	**0.920**
**H**	0.637	0.671	0.629	**0.977**	**0.988**	**0.997**		**0.988**	**0.995**	0.718	0.886
**T**	0.616	0.658	0.612	**0.979**	**0.989**	**0.998**	**0.998**		**0.981**	0.604	**0.932**
**W**	0.636	0.675	0.626	**0.973**	**0.988**	**0.992**	**0.996**	**0.995**		0.731	0.884
**P1**	**0.919**	**0.957**	**0.950**	0.474	0.516	0.588	0.611	0.607	0.602		0.378
**P2**	0.575	0.619	0.558	**0.955**	**0.952**	**0.951**	**0.952**	**0.954**	**0.952**	0.565	

Data in summer and winter are shown below and above the diagonal line, respectively. Data > 0.9 are written in bold.

**Table 3:** Measured and estimated concentrations of personal exposure to the highest-risk chemicals.

		P1 Exposure			P2 Exposure		
	Compound	Measured (µg/m^3^)	Estimated (µg/m^3^)	Estimated /Measured	Measured (µg/m^3^)	Estimated (µg/m^3^)	Estimated /Measured
**Summer**							
	acrolein	0.29	0.41	1.4	0.16	0.07	0.44
	*p*-dichlorobenzene	56	56	1.0	240	300	1.3
	acetaldehyde	71	53	0.75	20	12	0.60
**Winter**							
	acrolein	0.89	1.0	1.1	0.50	0.36	0.72
	nitrogen dioxide	240	280	1.2	52	53	1.0
	formic acid	140	160	1.1	43	33	0.77
	*p*-dichlorobenzene	70	76	1.1	320	460	1.4
	benzene	2.5	2.9	1.2	1.3	1.1	0.85

**Table 4:** Comparison of concentrations (µg/m^3^) of selected chemicals between the present study and previous studies.

Location	Year	Formaldehyde	Acetaldehyde	Chloroform	Benzene	Styrene	*p*-DCP	Note	Reference
New York, USA (Summer)	1999	19	11.0	1.7	1.5	0.5	6.1	median	Sax et al. (2004) [19]
New York, USA (Winter)	1999	12	14.0	2.6	3.6	1.0	8.9		
Shimizu, Japan (Summer)	2000	19	9.4	0.3	1.0	-	41.0	Geometric mean	Ohura et al. (2006) [20]
Shimizu, Japan (Winter)	2001	12	17.0	0.9	2.7	-	43.0		
All over Japan (Summer)	2012, 2013	27	13.0	0.0	1.0	-	4.3	median	Uchiyama et al. (2015) [10]
All over Japan (Winter)	2012–2014	11	15.0	0.4	1.7	-	1.4		
Beijing, China (non-heating)	2012	48	17.0	-	5.7	-	-	median	Duan et al. (2016) [21]
Beijing, China (heating)	2011	29	13.0	-	6.4	-	-		
Chiba, Japan (Summer)	2020	21–51	14–79	1.1–3.4	1.2–1.4	1.4–7.0	84–560	min.–max. in one apartment	This study
Chiba, Japan (Winter)	2021	16–35	15–40	1.2–2.9	2.0–3.6	11–130	88–850		

*p*-DCB: *p*-dichlorobenzene.

## Data Availability

The dataset generated during the current study are not publicly available but are available from the corresponding author on reasonable request.

## Supplementary Materials

The following are available online at https://www.mdpi.com/article/10.3390/ijerph182111511/s1, Figure S1: Outline of the procedure for PSD sampler, Figure S2: Concentrations of chemical compounds in the floor plan.
