# Supplementary material for: Spatial Variations of Indoor Air Chemicals in an Apartment Unit and Personal Exposure of Residents
Source: Int J Environ Res Public Health. 2021 Nov 1;18(21):11511. doi: 10.3390/ijerph182111511 (PMC8583336; doi:10.3390/ijerph182111511)
Supplement: Supplementary file 1 [file ijerph-18-11511-s001.zip › ijerph-1428716-supplementary.pdf]

Supplementary Information

Spatial Variations of Indoor Air Chemicals in an Apartment Unit and Personal Exposure of Residents

Hironari Sakamoto[1,2], Shigehisa Uchiyama[1†], Tomohiko Isobe[2], Naoki Kunugita[3], Hironao Ogura[1], Shoji F. Nakayama[2]

[1] Faculty and Graduate School of Engineering, Chiba University, 1-33 Yayoicho, Inage-ku, Chiba-shi, Chiba 263-8522, Japan

[2] Japan Environment and Children’s Study Program Office, National Institute for Environmental Studies, 16-2 Onogawa, Tsukuba-shi, Ibaraki 305-8506, Japan

[3] School of Health Sciences, University of Occupational and Environmental Health, 1-1 Iseigaoka, Yahatanishi-ku, Kitakyushu-shi, Fukuoka 807-8555, Japan

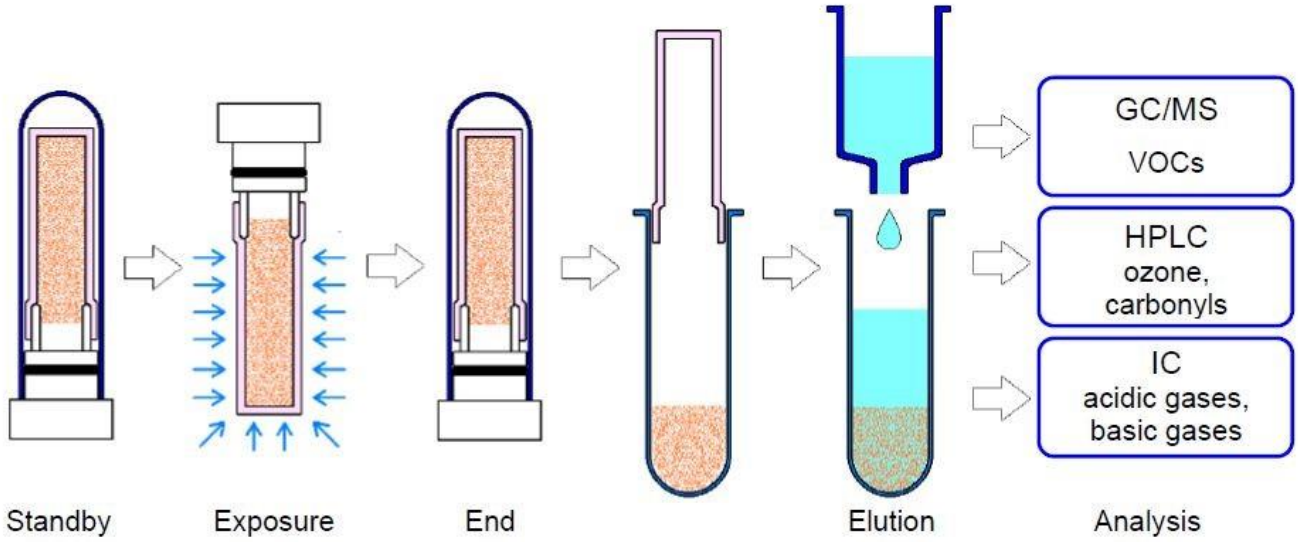

Figure S1. Outline of the procedure for DSD sampler

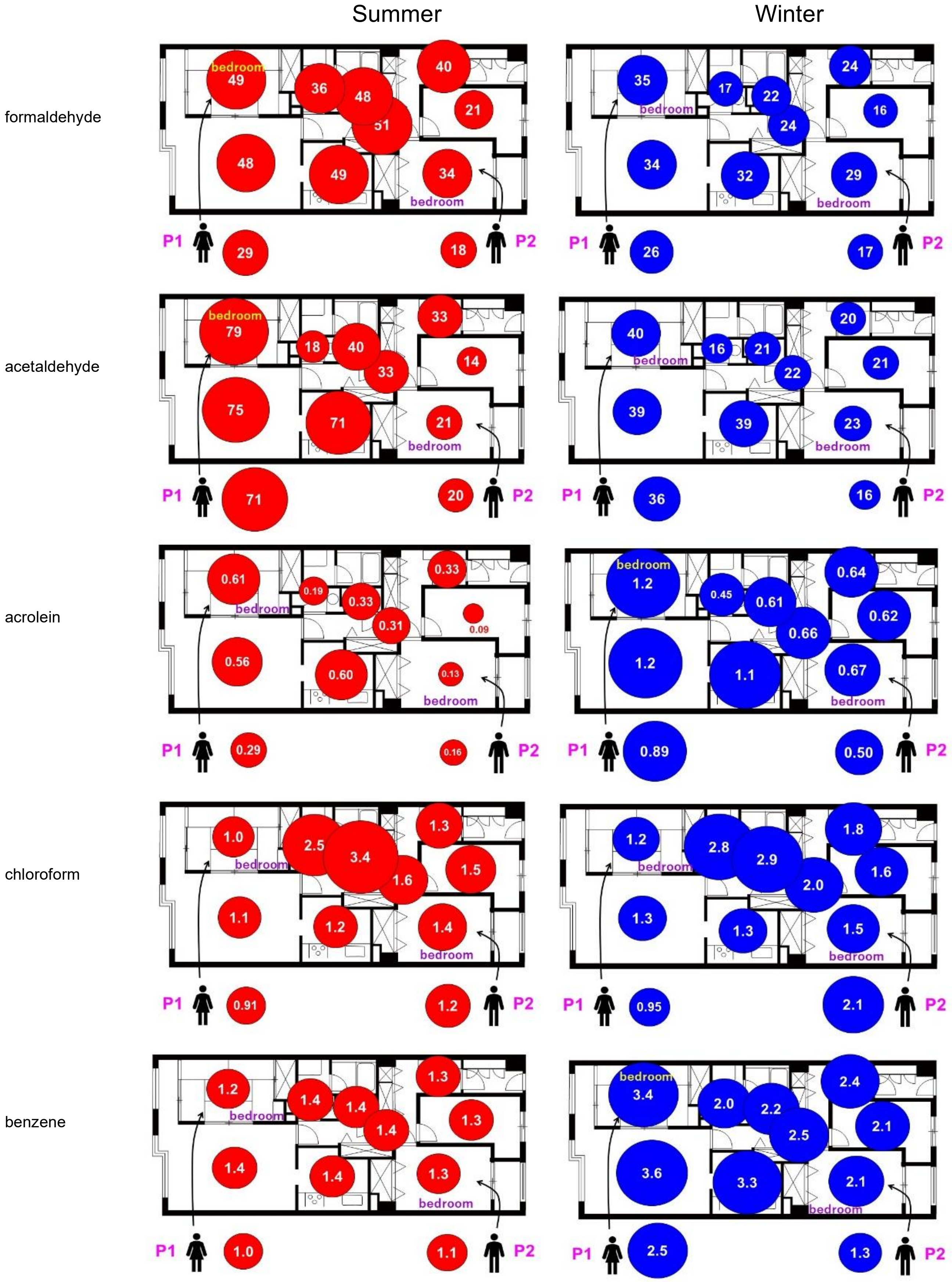

Figure S2. (a) Concentrations of chemical compounds in the floor plan.
Each circle area and number shows concentration (µg/m^3) at the space or the resident. The ratios of concentration to circle area differ by chemical compounds.

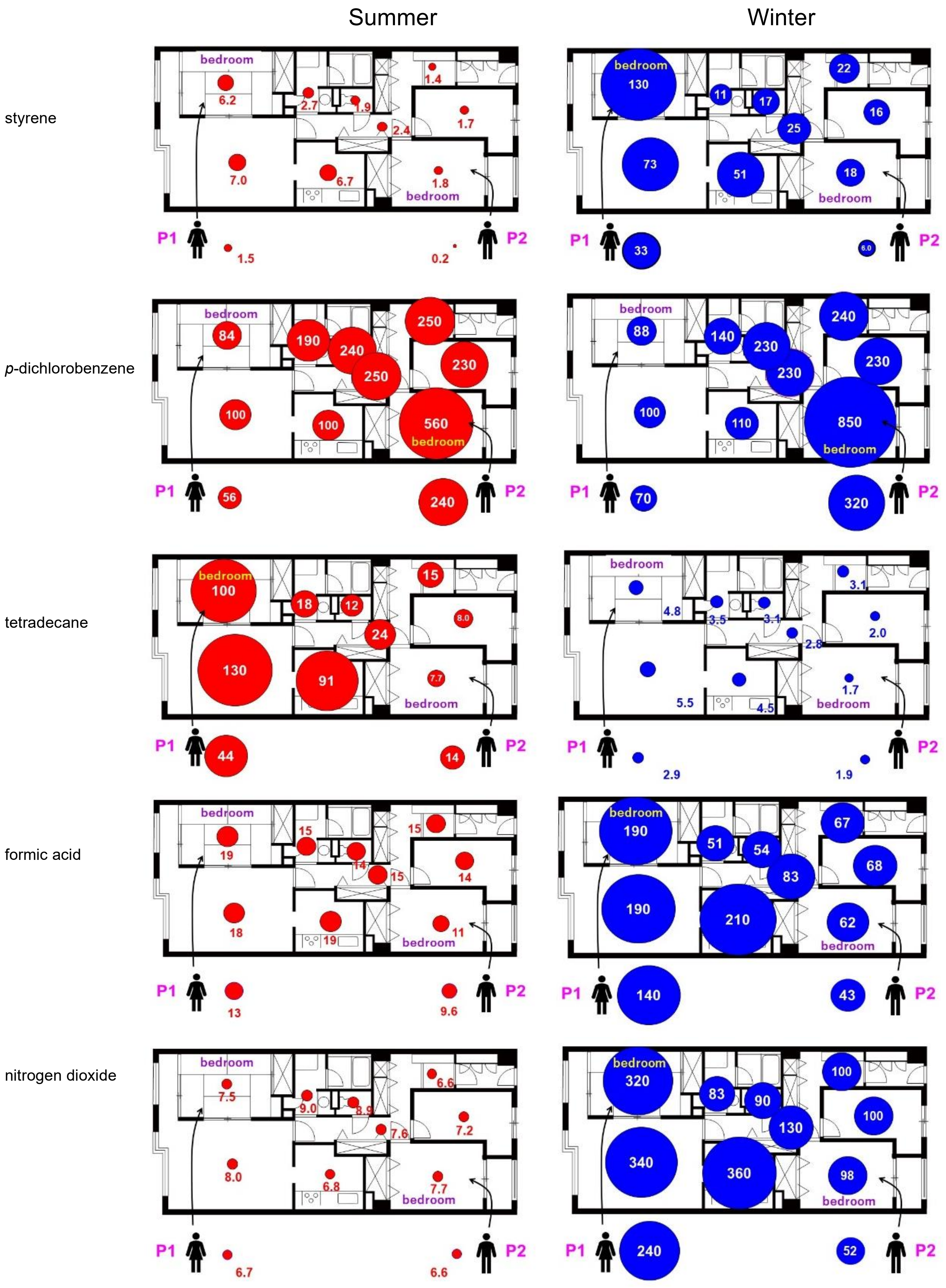

Figure S2. (b) Concentrations of chemical compounds in the floor plan (continued).
